# 4-Meth­oxy­benzamidinium bromide

**DOI:** 10.1107/S1600536812049872

**Published:** 2012-12-08

**Authors:** Simona Irrera, Gustavo Portalone

**Affiliations:** a Chemistry Department, "Sapienza" University of Rome, P.le A. Moro, 5, I-00185 Rome, Italy

## Abstract

The title salt, C_8_H_11_N_2_O^+^·Br^−^, was synthesized by the reaction between 4-meth­oxy­benzamidine (4-amidino­anisole) and hydro­bromic acid. In the cation, the amidinium group has two similar C—N bonds [1.304 (2) and 1.316 (2) Å], and its plane forms a dihedral angle of 31.08 (5)° with the benzene ring. The ions are associated in the crystal into a three-dimension hydrogen-bonded supra­molecular network featuring N—H^+^⋯Br^−^ inter­actions.

## Related literature

For the biological and pharmacological relevance of benzamidine, see: Powers & Harper (1999 ▶). For structural analysis of proton-transfer adducts containing mol­ecules of biological inter­est, see: Portalone (2011 ▶); Portalone & Irrera (2011 ▶). For the supra­molecular association in proton-transfer adducts containing benzamidinium cations, see: Portalone (2010 ▶, 2012 ▶); Irrera *et al.* (2012 ▶); Irrera & Portalone (2012*a* ▶,*b* ▶,*c* ▶,*d* ▶,*e* ▶). For hydrogen-bond motifs, see: Bernstein *et al.* (1995 ▶).
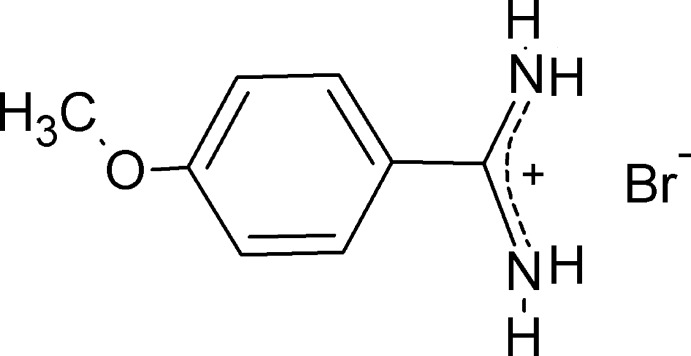


## Experimental

### 

#### Crystal data

C_8_H_11_N_2_O^+^·Br^−^
*M* *_r_* = 231.10
Orthorhombic, 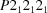

*a* = 7.5657 (6) Å
*b* = 10.8711 (7) Å
*c* = 11.5419 (7) Å
*V* = 949.29 (11) Å^3^
*Z* = 4
Mo *K*α radiation
μ = 4.29 mm^−1^
*T* = 298 K
0.18 × 0.12 × 0.10 mm

#### Data collection

Agilent Xcalibur Sapphire3 diffractometer
Absorption correction: multi-scan (*CrysAlis PRO*; Agilent, 2011 ▶) *T*_min_ = 0.513, *T*_max_ = 0.674
34724 measured reflections
3278 independent reflections
2903 reflections with *I* > 2σ(*I*)
*R*_int_ = 0.043

#### Refinement

*R*[*F* ^2^ > 2σ(*F* ^2^)] = 0.027
*wR*(*F* ^2^) = 0.055
*S* = 1.09
3278 reflections
126 parameters
H atoms treated by a mixture of independent and constrained refinement
Δρ_max_ = 0.22 e Å^−3^
Δρ_min_ = −0.31 e Å^−3^
Absolute structure: Flack (1983 ▶), 1387 Friedel pairs
Flack parameter: −0.002 (9)

### 

Data collection: *CrysAlis CCD* (Oxford Diffraction, 2006 ▶); cell refinement: *CrysAlis RED* (Oxford Diffraction, 2006 ▶); data reduction: *CrysAlis RED*; program(s) used to solve structure: *SIR97* (Altomare *et al.*, 1999 ▶); program(s) used to refine structure: *SHELXL97* (Sheldrick, 2008 ▶); molecular graphics: *WinGX* (Farrugia, 2012 ▶); software used to prepare material for publication: *WinGX* (Farrugia, 2012 ▶).

## Supplementary Material

Crystal structure: contains datablock(s) global, I. DOI: 10.1107/S1600536812049872/rz5032sup1.cif

Structure factors: contains datablock(s) I. DOI: 10.1107/S1600536812049872/rz5032Isup2.hkl

Additional supplementary materials:  crystallographic information; 3D view; checkCIF report

## Figures and Tables

**Table 1:** Hydrogen-bond geometry (Å, °)

*D*—H⋯*A*	*D*—H	H⋯*A*	*D*⋯*A*	*D*—H⋯*A*
N1—H1*A*⋯Br1	0.87 (3)	2.48 (3)	3.3163 (19)	159 (2)
N1—H1*B*⋯Br1^i^	0.88 (3)	2.49 (3)	3.3676 (19)	176 (2)
N2—H2*A*⋯Br1	0.95 (3)	2.65 (3)	3.4765 (17)	145 (2)
N2—H2*B*⋯Br1^ii^	0.78 (2)	2.70 (3)	3.4742 (17)	175 (2)

Symmetry codes: (i) 
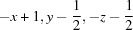
; (ii) 
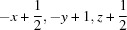
.

**Fig. 1.**
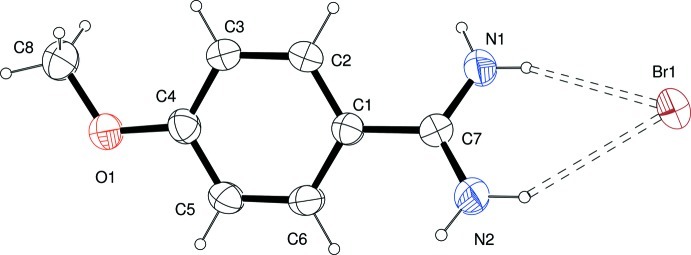
The asymmetric unit of the title compound, showing displacements ellipsoids drawn at the 50% probability level. H atoms are shown as small spheres of arbitrary radii and hydrogen bonds as dashed lines.

**Fig. 2.**
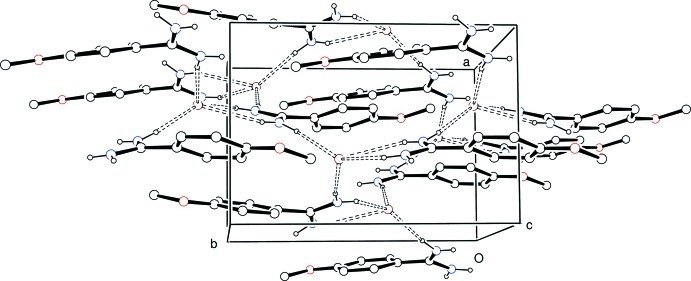
Crystal packing diagram of the title compound viewed approximately down c. All atoms are shown as small spheres of arbitrary radii. For clarity, H atoms not involved in hydrogen bonding (dashed lines) have been omitted.

## supplementary crystallographic information

### Comment

As part of our ongoing interest in systematic structural analysis of proton-transfer adducts containing molecules of biological interest (Portalone, 2011; Portalone & Irrera, 2011) this study reports the single-crystal structure of the title molecular salt, 4-methoxybenzamidinium bromide, (I), which was obtained by a reaction between 4-methoxybenzamidine (4-amidinoanisole) and hydrobromic acid in water solution. Benzamidine derivatives, which have shown strong biological and pharmacological activity (Powers & Harper, 1999), are being used in our group as bricks for supramolecular construction (Portalone, 2010; Portalone, 2012). Indeed, these molecules are strong Lewis base and their cations can be easily anchored onto numerous inorganic and organic anions and polyanions, largely because of the presence of four potential donor sites for hydrogen-bonding.

The asymmetric unit of (I) comprises one non-planar 4-methoxybenzamidinium cation and one bromide anion (Fig. 1).

In the cation the amidinium group forms a dihedral angle of 31.08 (5)° with the benzene ring, which is close to the values observed in protonated benzamidinium ions (23.2-30.4°; Portalone, 2010; Portalone, 2012). The lack of planarity in all these systems is obviously caused by steric hindrances between the H atoms of the aromatic ring and the amidine moiety. This conformation is rather common in benzamidinium-containing small molecule crystal structures, with the only exception of benzamidinium diliturate, where the benzamidinium cation is planar (Portalone, 2010). The pattern of bond lengths and bond angles of the 4-methoxybenzamidinium cation agrees with that reported in previous structural investigations (Portalone, 2010; Portalone, 2012; Irrera *et al.*, 2012; Irrera & Portalone, 2012*a*, 2012*b*, 2012*c*, 2012*d*, 2012*e*). In particular the amidinium group, true to one's expectations, features similar C—N bonds [1.304 (2) and 1.316 (2) Å], evidencing the delocalization of the π electrons and partial double-bond character.

Analysis of the crystal packing of (I), (Fig. 2), shows that each amidinium unit is bound to three bromide anions by four distinct weak N—H^+^···Br^-^ hydrogen bonds (N^+^···Br^-^ = 3.3163 (19)-3.4765 (17) Å; Table 1). The ion pairs of the asymmetric unit are joined by two N—H^+^···Br^-^ hydrogen bonds in ionic dimers, where Br^-^ anion acts as a bifurcated acceptor, thus generating an *R*^1^_2_(6) motif (Bernstein *et al.*, 1995). These subunits are then joined through the remaining N—H^+^···Br^-^ hydrogen bonds to adjacent Br^-^ anions leading to the formation of three-dimension hydrogen-bonded network.

### Experimental

4-Methoxybenzamidine (1 mmol, Fluka at 96% purity) was dissolved without further purification in 6 ml of hot water and heated under reflux for 6 h. While stirring, HBr (2 mol *L*^-1^) was added dropwise until pH reached 2. After cooling the solution to an ambient temperature, colourless crystals suitable for single-crystal X-ray diffraction were grown by slow evaporation of the solvent after four weeks.

### Refinement

All H atoms were identified in a difference Fourier map, but for refinement all C-bound H atoms were placed in calculated positions, with C—H = 0.93 Å (phenyl) and 0.96 Å (methyl), and refined as riding on their carrier atoms. The *U*_iso_ values were kept equal to 1.2*U*_eq_(C) or 1.5*U*_eq_(C) for methyl H atoms. The hydrogen atoms of the methyl group were allowed to rotate with a fixed angle around the C–C bond to best fit the experimental electron density [HFIX 137 in the *SHELX* program suite]. Positional and isotropic thermal parameters of H atoms of the amidinium group were freely refined, giving N—H distances in the range 0.78 (2)-0.95 (3) Å.

### Crystal data

**Table:**

C_8_H_11_N_2_O^+^·Br^−^	*F*(000) = 464
*M**_r_* = 231.10	*D*_x_ = 1.617 Mg m^−^^3^
Orthorhombic, *P*2_1_2_1_2_1_	Mo *K*α radiation, λ = 0.71069 Å
Hall symbol: P 2ac 2ab	Cell parameters from 10829 reflections
*a* = 7.5657 (6) Å	θ = 3.2–32.5°
*b* = 10.8711 (7) Å	µ = 4.29 mm^−^^1^
*c* = 11.5419 (7) Å	*T* = 298 K
*V* = 949.29 (11) Å^3^	Tablets, colourless
*Z* = 4	0.18 × 0.12 × 0.10 mm

### Data collection

**Table:**

Agilent Xcalibur Sapphire3 diffractometer	3278 independent reflections
Radiation source: Enhance (Mo) X-ray Source	2903 reflections with *I* > 2σ(*I*)
Graphite monochromator	*R*_int_ = 0.043
Detector resolution: 16.0696 pixels mm^-1^	θ_max_ = 32.0°, θ_min_ = 3.2°
ω and φ scans	*h* = −11→11
Absorption correction: multi-scan (*CrysAlis PRO*; Agilent, 2011)	*k* = −16→16
*T*_min_ = 0.513, *T*_max_ = 0.674	*l* = −17→17
34724 measured reflections	

### Refinement

**Table:**

Refinement on *F*^2^	Secondary atom site location: difference Fourier map
Least-squares matrix: full	Hydrogen site location: inferred from neighbouring sites
*R*[*F*^2^ > 2σ(*F*^2^)] = 0.027	H atoms treated by a mixture of independent and constrained refinement
*wR*(*F*^2^) = 0.055	*w* = 1/[σ^2^(*F*_o_^2^) + (0.0236*P*)^2^ + 0.1235*P*] where *P* = (*F*_o_^2^ + 2*F*_c_^2^)/3
*S* = 1.09	(Δ/σ)_max_ < 0.001
3278 reflections	Δρ_max_ = 0.22 e Å^−^^3^
126 parameters	Δρ_min_ = −0.31 e Å^−^^3^
0 restraints	Absolute structure: Flack (1983), 1387 Friedel pairs
Primary atom site location: structure-invariant direct methods	Flack parameter: −0.002 (9)

### Special details

**Table:**

Geometry. All s.u.'s (except the s.u. in the dihedral angle between two l.s. planes) are estimated using the full covariance matrix. The cell s.u.'s are taken into account individually in the estimation of s.u.'s in distances, angles and torsion angles; correlations between s.u.'s in cell parameters are only used when they are defined by crystal symmetry. An approximate (isotropic) treatment of cell s.u.'s is used for estimating s.u.'s involving l.s. planes.
Refinement. Refinement of *F*^2^ against ALL reflections. The weighted *R*-factor *wR* and goodness of fit *S* are based on *F*^2^, conventional *R*-factors *R* are based on *F*, with *F* set to zero for negative *F*^2^. The threshold expression of *F*^2^ > 2σ(*F*^2^) is used only for calculating *R*-factors(gt) *etc*. and is not relevant to the choice of reflections for refinement. *R*-factors based on *F*^2^ are statistically about twice as large as those based on *F*, and *R*- factors based on ALL data will be even larger.

### Fractional atomic coordinates and isotropic or equivalent isotropic displacement parameters (Å^2^)

**Table:**

	*x*	*y*	*z*	*U*_iso_*/*U*_eq_	
Br1	0.35106 (3)	0.611694 (17)	−0.195955 (16)	0.04325 (6)	
O1	0.3480 (2)	−0.16213 (12)	0.17351 (11)	0.0435 (3)	
N1	0.4313 (3)	0.31784 (17)	−0.13950 (15)	0.0431 (4)	
H1A	0.431 (3)	0.392 (3)	−0.169 (2)	0.056 (7)*	
H1B	0.484 (3)	0.261 (3)	−0.181 (2)	0.062 (8)*	
N2	0.3375 (3)	0.40022 (16)	0.02982 (15)	0.0440 (4)	
H2A	0.329 (3)	0.480 (2)	−0.003 (2)	0.060 (7)*	
H2B	0.300 (3)	0.394 (3)	0.092 (2)	0.054 (7)*	
C1	0.3765 (2)	0.18004 (16)	0.01954 (14)	0.0312 (3)	
C2	0.3378 (3)	0.07914 (17)	−0.04827 (15)	0.0387 (4)	
H2	0.3177	0.0898	−0.1271	0.046*	
C3	0.3284 (3)	−0.03770 (17)	−0.00107 (16)	0.0407 (4)	
H3	0.3034	−0.1052	−0.0477	0.049*	
C4	0.3568 (3)	−0.05267 (15)	0.11722 (15)	0.0347 (3)	
C5	0.3987 (2)	0.04789 (17)	0.18527 (16)	0.0383 (4)	
H5	0.4210	0.0370	0.2638	0.046*	
C6	0.4076 (2)	0.16333 (18)	0.13810 (16)	0.0364 (4)	
H6	0.4344	0.2306	0.1848	0.044*	
C7	0.3823 (2)	0.30343 (17)	−0.03201 (15)	0.0319 (4)	
C8	0.3064 (3)	−0.26858 (18)	0.1078 (2)	0.0521 (6)	
H8A	0.1936	−0.2578	0.0711	0.078*	
H8B	0.3021	−0.3388	0.1581	0.078*	
H8C	0.3955	−0.2811	0.0498	0.078*	

### Atomic displacement parameters (Å^2^)

**Table:**

	*U*^11^	*U*^22^	*U*^33^	*U*^12^	*U*^13^	*U*^23^
Br1	0.05673 (11)	0.03755 (9)	0.03548 (8)	−0.00280 (10)	−0.00105 (9)	0.01017 (8)
O1	0.0614 (8)	0.0328 (6)	0.0364 (7)	0.0010 (7)	−0.0039 (7)	0.0042 (5)
N1	0.0643 (11)	0.0313 (8)	0.0338 (8)	0.0052 (8)	0.0086 (8)	0.0021 (7)
N2	0.0634 (10)	0.0317 (8)	0.0368 (8)	0.0032 (10)	0.0107 (8)	−0.0013 (7)
C1	0.0340 (9)	0.0304 (8)	0.0291 (7)	0.0011 (7)	0.0007 (7)	0.0000 (6)
C2	0.0536 (11)	0.0369 (9)	0.0255 (7)	−0.0042 (9)	−0.0032 (8)	−0.0002 (6)
C3	0.0581 (13)	0.0329 (8)	0.0311 (8)	−0.0050 (9)	−0.0029 (9)	−0.0035 (7)
C4	0.0385 (8)	0.0324 (8)	0.0332 (8)	0.0036 (9)	0.0004 (9)	0.0038 (6)
C5	0.0489 (10)	0.0387 (9)	0.0272 (8)	0.0020 (7)	−0.0061 (8)	0.0017 (7)
C6	0.0459 (10)	0.0335 (9)	0.0298 (8)	0.0018 (8)	−0.0040 (7)	−0.0055 (7)
C7	0.0331 (9)	0.0312 (8)	0.0313 (8)	−0.0008 (7)	−0.0005 (7)	−0.0012 (6)
C8	0.0759 (17)	0.0332 (10)	0.0474 (11)	0.0008 (10)	0.0045 (11)	0.0001 (8)

### Geometric parameters (Å, º)

**Table:**

O1—C4	1.357 (2)	C2—C3	1.384 (3)
O1—C8	1.419 (2)	C2—H2	0.9300
N1—C7	1.304 (2)	C3—C4	1.392 (2)
N1—H1A	0.87 (3)	C3—H3	0.9300
N1—H1B	0.88 (3)	C4—C5	1.383 (3)
N2—C7	1.316 (2)	C5—C6	1.370 (3)
N2—H2A	0.95 (3)	C5—H5	0.9300
N2—H2B	0.78 (2)	C6—H6	0.9300
C1—C2	1.379 (2)	C8—H8A	0.9600
C1—C6	1.400 (2)	C8—H8B	0.9600
C1—C7	1.468 (2)	C8—H8C	0.9600
			
C4—O1—C8	118.05 (14)	O1—C4—C3	124.37 (16)
C7—N1—H1A	118.6 (16)	C5—C4—C3	120.01 (16)
C7—N1—H1B	124.6 (17)	C6—C5—C4	120.66 (16)
H1A—N1—H1B	116 (2)	C6—C5—H5	119.7
C7—N2—H2A	122.2 (16)	C4—C5—H5	119.7
C7—N2—H2B	122 (2)	C5—C6—C1	119.93 (17)
H2A—N2—H2B	115 (3)	C5—C6—H6	120.0
C2—C1—C6	119.15 (17)	C1—C6—H6	120.0
C2—C1—C7	120.22 (15)	N1—C7—N2	119.54 (18)
C6—C1—C7	120.62 (16)	N1—C7—C1	120.25 (16)
C1—C2—C3	121.18 (16)	N2—C7—C1	120.21 (16)
C1—C2—H2	119.4	O1—C8—H8A	109.5
C3—C2—H2	119.4	O1—C8—H8B	109.5
C2—C3—C4	119.05 (16)	H8A—C8—H8B	109.5
C2—C3—H3	120.5	O1—C8—H8C	109.5
C4—C3—H3	120.5	H8A—C8—H8C	109.5
O1—C4—C5	115.62 (15)	H8B—C8—H8C	109.5
			
C6—C1—C2—C3	0.4 (3)	C3—C4—C5—C6	1.9 (3)
C7—C1—C2—C3	−178.8 (2)	C4—C5—C6—C1	−0.8 (3)
C1—C2—C3—C4	0.7 (3)	C2—C1—C6—C5	−0.3 (3)
C8—O1—C4—C5	−179.67 (18)	C7—C1—C6—C5	178.87 (17)
C8—O1—C4—C3	−0.2 (3)	C2—C1—C7—N1	−31.3 (3)
C2—C3—C4—O1	178.8 (2)	C6—C1—C7—N1	149.52 (19)
C2—C3—C4—C5	−1.8 (3)	C2—C1—C7—N2	148.4 (2)
O1—C4—C5—C6	−178.63 (18)	C6—C1—C7—N2	−30.7 (3)

### Hydrogen-bond geometry (Å, º)

**Table:**

*D*—H···*A*	*D*—H	H···*A*	*D*···*A*	*D*—H···*A*
N1—H1*A*···Br1	0.87 (3)	2.48 (3)	3.3163 (19)	159 (2)
N1—H1*B*···Br1^i^	0.88 (3)	2.49 (3)	3.3676 (19)	176 (2)
N2—H2*A*···Br1	0.95 (3)	2.65 (3)	3.4765 (17)	145 (2)
N2—H2*B*···Br1^ii^	0.78 (2)	2.70 (3)	3.4742 (17)	175 (2)

Symmetry codes: (i) −*x*+1, *y*−1/2, −*z*−1/2; (ii) −*x*+1/2, −*y*+1, *z*+1/2.
